# Dichloridobis(3-chloro­pyridine-κ*N*)zinc

**DOI:** 10.1107/S1600536811020447

**Published:** 2011-06-11

**Authors:** Yan-Hui Liu, Lin Xu, Dong-Mei Dai, Jian-Wei Zou

**Affiliations:** a Department of Chemical and Biological Engineering, Zhejiang University, Hangzhou, Zhejiang 310027, People’s Republic of China; b Department of Chemistry, Zhejiang University, Hangzhou 310027, People’s Republic of China; c Ningbo Institute of Technology, Zhejiang University, Ningbo, Zhejiang 315100, People’s Republic of China

## Abstract

In the crystal structure of the title compound, [ZnCl_2_(C_5_H_4_ClN)_2_], discrete complex mol­ecules are found in which the Zn^II^ cations are coordinated by two chloride anions and the N atoms of the two 3-chloro­pyridine ligands within a slightly distorted tetra­hedron. Moreover, inter­molecular C—Cl⋯Cl—C halogen inter­actions (Cl⋯Cl = 3.442 Å) are found between the building blocks.

## Related literature

For the background of this work, see: Bertani *et al. *(2010 ▶); Metrangolo & Resnati (2001 ▶); Leininger *et al.* (2000 ▶); Lommerse *et al.* (1996 ▶). For related structures, see: Bhosekar *et al.* (2008 ▶); Wriedt *et al.* (2009 ▶).
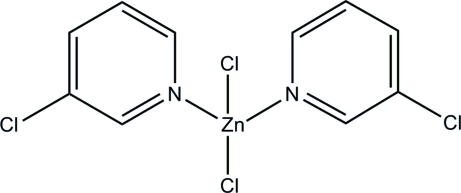


## Experimental

### 

#### Crystal data

[ZnCl_2_(C_5_H_4_ClN)_2_]
*M* *_r_* = 363.35
Triclinic, 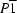

*a* = 7.3429 (15) Å
*b* = 7.9220 (16) Å
*c* = 13.259 (3) Å
α = 95.17 (3)°
β = 91.14 (3)°
γ = 117.37 (3)°
*V* = 680.5 (2) Å^3^
*Z* = 2
Mo *K*α radiation
μ = 2.57 mm^−1^
*T* = 298 K
0.44 × 0.42 × 0.19 mm

#### Data collection

Siemens CCD diffractometer
Absorption correction: multi-scan (*SADABS*; Bruker, 2002 ▶) *T*_min_ = 0.398, *T*_max_ = 0.641
5839 measured reflections
2640 independent reflections
2066 reflections with *I* > 2σ(*I*)
*R*_int_ = 0.044

#### Refinement

*R*[*F* ^2^ > 2σ(*F* ^2^)] = 0.042
*wR*(*F* ^2^) = 0.147
*S* = 1.16
2640 reflections
154 parameters
H-atom parameters constrained
Δρ_max_ = 0.82 e Å^−3^
Δρ_min_ = −1.24 e Å^−3^

### 

Data collection: *XSCANS* (Siemens, 1994 ▶); cell refinement: *XSCANS*; data reduction: *SHELXTL* (Sheldrick, 2008 ▶); program(s) used to solve structure: *SHELXS97* (Sheldrick, 2008 ▶); program(s) used to refine structure: *SHELXL97* (Sheldrick, 2008 ▶); molecular graphics: *SHELXTL*; software used to prepare material for publication: *SHELXTL* and *publCIF* (Westrip, 2010 ▶).

## Supplementary Material

Crystal structure: contains datablock(s) I, global. DOI: 10.1107/S1600536811020447/nc2231sup1.cif

Structure factors: contains datablock(s) I. DOI: 10.1107/S1600536811020447/nc2231Isup2.hkl

Additional supplementary materials:  crystallographic information; 3D view; checkCIF report

## Figures and Tables

**Fig. 1.**
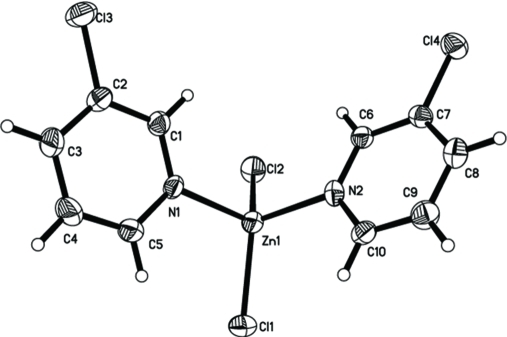
Crystal structure of the title compound with labeling and displacement elliposids drawn at the 30% probability level.

**Fig. 2.**
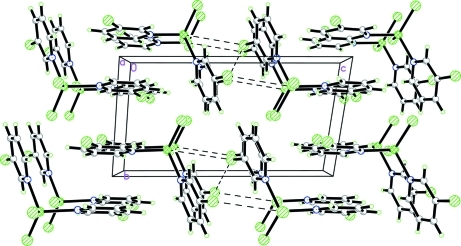
Crystal structure of the title compound with view along the c-axis and C—Cl···Cl interactions shown as dashed lines.

## supplementary crystallographic information

### Comment

Halogen interactions as one weak noncovalent interaction, is of importance in e.g. crystal engineering and molecular recognition processes (Metrangolo & Resnati, 2001). Such interactions are widely found in various organometallic coordination compounds like e.g. in coordination compounds built up on multidentate ligands with pyridine groups which generate networks with a variety of special functions ( Leininger *et al.*, 2000 and Bertani *et al.*, 2010).

As a part of our project on halogen halogen interactions the tile compound was prepared and characterized by single crystal X-ray diffraction. In the crystal structure of the title compound discrete complexes are found in which each zinc(II) cation is coordinated by two 3-chloropyridine ligands and two chloride anions. The coordination environment around the Zn cations consists of slightly distorted tetrahedra, which is typical for such complexes ( Bhosekar *et al.*, 2008; Wriedt *et al.*, 2009). The crystal structure is characterized by intermolecular C—Cl···Cl—C interactions with Cl···Cl separations less than the sum of Van der Waals radii (Lommerse, *et al.*, 1996).

### Experimental

Zinc(II) chloride (1 mmol) dissolved in 10 mL of ethanol, was added dropwise to a stirred solution of 3-chloropyridine (1 mmol) in 10 mL of ethanol. Subsequently, the mixture was refluxed for 2 h, and the resulting solution was further concentrated by the rotary evaporation at 40 Celsius degree. Finally, the concentrated solution was left to slowly evaporate at room temperature until the crystal formed.

### Refinement

All H atoms were placed in calculated positions and allowed to ride on their parent atoms at distances of 0.93Å with isotropic displacement parameters 1.2 times Ueq of the parent atoms.

### Crystal data

**Table:**

[ZnCl_2_(C_5_H_4_ClN)_2_]	*Z* = 2
*M**_r_* = 363.35	*F*(000) = 360
Triclinic, *P*1	*D*_x_ = 1.773 Mg m^−^^3^
Hall symbol: -P 1	Mo *K*α radiation, λ = 0.71073 Å
*a* = 7.3429 (15) Å	Cell parameters from 2456 reflections
*b* = 7.9220 (16) Å	θ = 2.1–19.6°
*c* = 13.259 (3) Å	µ = 2.57 mm^−^^1^
α = 95.17 (3)°	*T* = 298 K
β = 91.14 (3)°	Prism, colorless
γ = 117.37 (3)°	0.44 × 0.42 × 0.19 mm
*V* = 680.5 (2) Å^3^	

### Data collection

**Table:**

Bruker P4 diffractometer	2640 independent reflections
Radiation source: fine-focus sealed tube	2066 reflections with *I* > 2σ(*I*)
graphite	*R*_int_ = 0.044
ω scans	θ_max_ = 26.0°, θ_min_ = 3.1°
Absorption correction: multi-scan (*SADABS*; Bruker, 2002)	*h* = −9→9
*T*_min_ = 0.398, *T*_max_ = 0.641	*k* = −9→9
5839 measured reflections	*l* = −16→13

### Refinement

**Table:**

Refinement on *F*^2^	Primary atom site location: structure-invariant direct methods
Least-squares matrix: full	Secondary atom site location: difference Fourier map
*R*[*F*^2^ > 2σ(*F*^2^)] = 0.042	Hydrogen site location: inferred from neighbouring sites
*wR*(*F*^2^) = 0.147	H-atom parameters constrained
*S* = 1.16	*w* = 1/[σ^2^(*F*_o_^2^) + (0.051*P*)^2^ + 1.6856*P*] where *P* = (*F*_o_^2^ + 2*F*_c_^2^)/3
2640 reflections	(Δ/σ)_max_ < 0.001
154 parameters	Δρ_max_ = 0.82 e Å^−^^3^
0 restraints	Δρ_min_ = −1.24 e Å^−^^3^

### Special details

**Table:**

Geometry. All esds (except the esd in the dihedral angle between two l.s. planes) are estimated using the full covariance matrix. The cell esds are taken into account individually in the estimation of esds in distances, angles and torsion angles; correlations between esds in cell parameters are only used when they are defined by crystal symmetry. An approximate (isotropic) treatment of cell esds is used for estimating esds involving l.s. planes.
Refinement. Refinement of F^2^ against ALL reflections. The weighted R-factor wR and goodness of fit S are based on F^2^, conventional R-factors R are based on F, with F set to zero for negative F^2^. The threshold expression of F^2^ > 2sigma(F^2^) is used only for calculating R-factors(gt) etc. and is not relevant to the choice of reflections for refinement. R-factors based on F^2^ are statistically about twice as large as those based on F, and R- factors based on ALL data will be even larger.

### Fractional atomic coordinates and isotropic or equivalent isotropic displacement parameters (Å^2^)

**Table:**

	*x*	*y*	*z*	*U*_iso_*/*U*_eq_	
Zn1	0.13903 (9)	0.76409 (9)	0.24334 (5)	0.0394 (2)	
Cl4	0.9010 (2)	1.1496 (2)	0.46968 (12)	0.0522 (4)	
Cl2	0.1747 (3)	0.5228 (2)	0.29582 (12)	0.0544 (4)	
Cl3	0.6030 (3)	0.6881 (3)	−0.08020 (13)	0.0623 (5)	
Cl1	−0.1516 (2)	0.7837 (3)	0.24273 (13)	0.0620 (5)	
N1	0.2137 (7)	0.7713 (6)	0.0913 (3)	0.0399 (10)	
N2	0.3755 (7)	1.0102 (6)	0.3193 (3)	0.0373 (10)	
C7	0.6947 (7)	1.1654 (7)	0.4135 (4)	0.0357 (11)	
C6	0.5425 (8)	1.0075 (8)	0.3607 (4)	0.0394 (12)	
H6A	0.5536	0.8948	0.3529	0.047*	
C2	0.4034 (9)	0.7317 (8)	−0.0417 (4)	0.0417 (12)	
C1	0.3685 (8)	0.7401 (7)	0.0598 (4)	0.0404 (12)	
H1A	0.4533	0.7241	0.1069	0.048*	
C10	0.3626 (9)	1.1745 (8)	0.3302 (4)	0.0404 (12)	
H10A	0.2466	1.1768	0.3024	0.049*	
C8	0.6861 (9)	1.3362 (8)	0.4244 (5)	0.0483 (14)	
H8A	0.7912	1.4458	0.4597	0.058*	
C3	0.2782 (10)	0.7512 (9)	−0.1128 (4)	0.0530 (15)	
H3A	0.2975	0.7388	−0.1817	0.064*	
C5	0.0957 (9)	0.7987 (9)	0.0231 (4)	0.0488 (14)	
H5A	−0.0088	0.8248	0.0457	0.059*	
C9	0.5139 (10)	1.3371 (8)	0.3805 (5)	0.0510 (15)	
H9A	0.5021	1.4495	0.3856	0.061*	
C4	0.1231 (10)	0.7898 (11)	−0.0794 (5)	0.0623 (18)	
H4A	0.0389	0.8094	−0.1251	0.075*	

### Atomic displacement parameters (Å^2^)

**Table:**

	*U*^11^	*U*^22^	*U*^33^	*U*^12^	*U*^13^	*U*^23^
Zn1	0.0408 (4)	0.0457 (4)	0.0344 (4)	0.0230 (3)	0.0014 (3)	0.0013 (3)
Cl4	0.0480 (8)	0.0630 (9)	0.0488 (9)	0.0289 (7)	−0.0058 (6)	0.0059 (7)
Cl2	0.0670 (10)	0.0490 (8)	0.0522 (9)	0.0309 (7)	−0.0005 (7)	0.0082 (6)
Cl3	0.0603 (10)	0.0855 (12)	0.0581 (10)	0.0463 (9)	0.0203 (8)	0.0154 (8)
Cl1	0.0484 (8)	0.0895 (12)	0.0586 (10)	0.0424 (9)	0.0047 (7)	−0.0018 (8)
N1	0.048 (3)	0.048 (2)	0.030 (2)	0.028 (2)	0.0003 (19)	0.0024 (18)
N2	0.043 (2)	0.039 (2)	0.028 (2)	0.018 (2)	0.0044 (18)	0.0022 (17)
C7	0.029 (2)	0.040 (3)	0.035 (3)	0.013 (2)	0.000 (2)	0.006 (2)
C6	0.047 (3)	0.049 (3)	0.032 (3)	0.029 (3)	0.006 (2)	0.008 (2)
C2	0.047 (3)	0.044 (3)	0.041 (3)	0.026 (3)	0.010 (2)	0.006 (2)
C1	0.046 (3)	0.044 (3)	0.039 (3)	0.029 (3)	−0.002 (2)	0.003 (2)
C10	0.047 (3)	0.050 (3)	0.035 (3)	0.031 (3)	0.006 (2)	0.007 (2)
C8	0.049 (3)	0.039 (3)	0.046 (3)	0.014 (3)	−0.004 (3)	−0.005 (2)
C3	0.053 (3)	0.072 (4)	0.029 (3)	0.025 (3)	0.005 (2)	0.005 (3)
C5	0.049 (3)	0.068 (4)	0.041 (3)	0.037 (3)	0.002 (3)	0.005 (3)
C9	0.060 (4)	0.043 (3)	0.055 (4)	0.029 (3)	−0.001 (3)	0.003 (3)
C4	0.060 (4)	0.101 (5)	0.037 (3)	0.046 (4)	−0.002 (3)	0.011 (3)

### Geometric parameters (Å, °)

**Table:**

Zn1—N2	2.072 (4)	C2—C3	1.372 (8)
Zn1—N1	2.098 (4)	C2—C1	1.376 (8)
Zn1—Cl1	2.2099 (17)	C1—H1A	0.9300
Zn1—Cl2	2.2130 (16)	C10—C9	1.356 (8)
Cl4—C7	1.736 (5)	C10—H10A	0.9300
Cl3—C2	1.730 (6)	C8—C9	1.385 (9)
N1—C1	1.336 (7)	C8—H8A	0.9300
N1—C5	1.340 (7)	C3—C4	1.379 (9)
N2—C10	1.342 (7)	C3—H3A	0.9300
N2—C6	1.344 (7)	C5—C4	1.378 (9)
C7—C6	1.352 (7)	C5—H5A	0.9300
C7—C8	1.378 (8)	C9—H9A	0.9300
C6—H6A	0.9300	C4—H4A	0.9300
			
N2—Zn1—N1	104.62 (18)	N1—C1—C2	120.5 (5)
N2—Zn1—Cl1	110.33 (14)	N1—C1—H1A	119.7
N1—Zn1—Cl1	104.81 (14)	C2—C1—H1A	119.7
N2—Zn1—Cl2	105.93 (14)	N2—C10—C9	121.9 (5)
N1—Zn1—Cl2	105.50 (13)	N2—C10—H10A	119.1
Cl1—Zn1—Cl2	124.03 (8)	C9—C10—H10A	119.1
C1—N1—C5	119.1 (5)	C7—C8—C9	117.0 (5)
C1—N1—Zn1	122.1 (4)	C7—C8—H8A	121.5
C5—N1—Zn1	118.7 (4)	C9—C8—H8A	121.5
C10—N2—C6	118.7 (5)	C2—C3—C4	118.2 (6)
C10—N2—Zn1	120.6 (4)	C2—C3—H3A	120.9
C6—N2—Zn1	120.7 (4)	C4—C3—H3A	120.9
C6—C7—C8	121.0 (5)	N1—C5—C4	122.5 (6)
C6—C7—Cl4	119.0 (4)	N1—C5—H5A	118.7
C8—C7—Cl4	120.0 (4)	C4—C5—H5A	118.7
N2—C6—C7	121.3 (5)	C10—C9—C8	120.1 (5)
N2—C6—H6A	119.4	C10—C9—H9A	120.0
C7—C6—H6A	119.4	C8—C9—H9A	120.0
C3—C2—C1	120.9 (5)	C5—C4—C3	118.6 (6)
C3—C2—Cl3	119.6 (5)	C5—C4—H4A	120.7
C1—C2—Cl3	119.4 (4)	C3—C4—H4A	120.7
